# Supplementary material for: Specificity and Origin of the Stability of the Sr Isotopic Ratio in Champagne Wines
Source: Molecules. 2021 Aug 23;26(16):5104. doi: 10.3390/molecules26165104 (PMC8400214; doi:10.3390/molecules26165104)
Supplement: Supplementary file 1 [file molecules-26-05104-s001.zip › molecules-1272748-supplementary.pdf]

Supplementary Material:

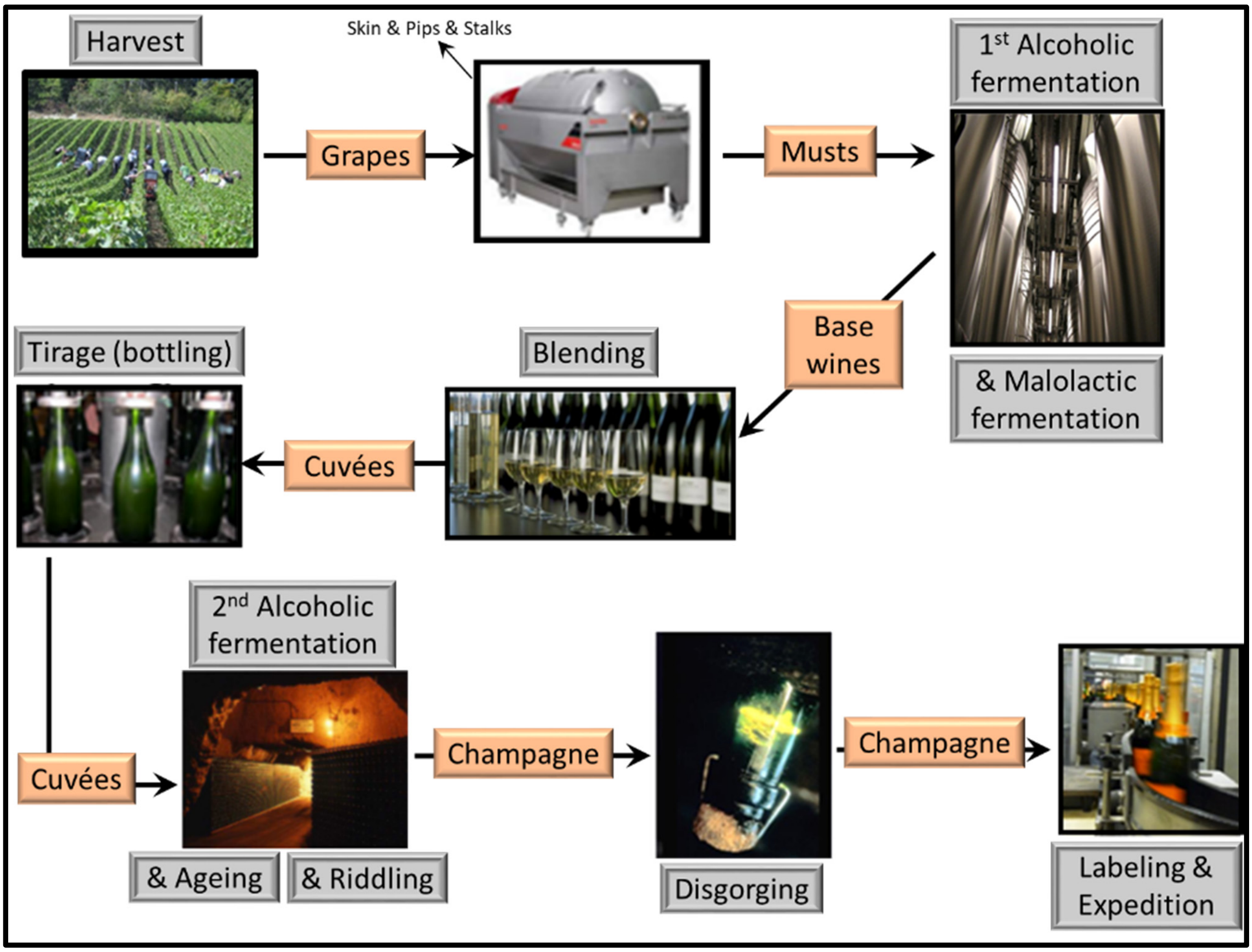

Figure S1: Traditional and typical steps for the elaboration process of the champagne – “Méthode traditionelle”.

**2 types of samples**		**Number of Samples**			
		**Grape**	**Must**	**Base wine**	**Champagne**
**Whole AOC Champagne**		0	25	11	39
Years		/	2018	2019	1983 to 2016
Blending		/	/	Yes	Yes
Area		/	from 3 departments		
**Small Plots**	**A**	1	1	1	1
	**B**	1	1	1	1
Years		2019	2019	2019	2000
Blending		No			
Area		A = 1,8 hectare ; B = 0,7 hectare			

Table S1: Description of the different samples analyzed for this study.

MC-ICP-MS	
**General Parameters**	
Plasma gas flow rate ($L.min^{-1}$)	13
Auxiliary flow rate ($L.min^{-1}$)	0,8
RF power (W)	1300
Acceleration voltage (V)	6000
Mass resolution	Low
**Sr measurments**	
Plasma mode	Wet
Sample Introduction	200 $\mu L.min^{-1}$ nebulizer + cyclonic spray chamber
Interface sampler cone	Ni, type A
Interface skimmer cone	Ni, type A
Sensitivity ($V_{88Sr}/ppm_{Total\ Sr}$)	34 to 44
Aquisition	600 s
Uptake flow rate ($mL.min^{-1}$)	0,2
Blank	OPZ aquired during 60s

Table S2: Operating conditions for the MC-ICP-MS for Sr isotopic analysis.
